# Ethylene treatment of “Maekawa‐Jiro” persimmon affects peel characteristics and consequently, enables boil‐peeling

**DOI:** 10.1002/fsn3.2214

**Published:** 2021-03-17

**Authors:** Satoru Murakami, Kazuki Yamaguchi, Nozomi Hashimoto

**Affiliations:** ^1^ Shizuoka Research Institute of Agriculture and Forestry Fruit Tree Research Center Mobata, Shimizu‐ku Shizuoka Japan

**Keywords:** ethylene, fruit, peeling, persimmon, polygalacturonase

## Abstract

In a previous study, we reported that ethylene treatment facilitated boil‐peeling in persimmons and in several other fruits; however, the mechanism underlying the facilitating effect of ethylene was not examined in detail. Thus, in this study, we investigated the effect of ethylene treatment on the peel characteristics of persimmons, that facilitated boil‐peeling, using chemical, genomic, and histochemistry analyses. The results of the study showed that the ethylene‐related genes, *DK‐ACS1* and *DK‐ACO2*, and the pectinase‐active gene *DKPG* were not expressed, even though a minor increase in ethylene generation was observed after ethylene treatment. Conversely, significant accumulation of toluidine blue O and ruthenium red dyes were observed in the sarcocarp and exocarp of the fruits, indicating an increase in the quantity of polysaccharides, including pectic substances, at the site. The results also indicate that the increased cellulase activity observed in the pericarp of the fruits may be due to the aging of the fruits, and not necessarily as a result of ethylene treatment. Furthermore, ethylene treatment increased the quantity of polysaccharides, including pectic substances, directly below the pericarp, which caused the dissolution of the site, resulting in peeling. This study provides new insights on the effect of ethylene on boil‐peeling in persimmons and provides a foundation for future research studying the effect of heat treatment in the peeling of fruits or tomato.

## Graphical abstract

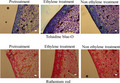

## 1 INTRODUCTION

Peeling is an important step in the preparation of most fruits and vegetables. In food processing, peeling of fruits and vegetables is performed using knives and chemicals (i.e., lye) and also by mechanical methods (i.e., abrasion), freezing, steaming, and boil‐peeling. Each of these conventional methods possesses their unique advantages and disadvantages for fruit quality and production cost. Boil‐peeling is a common method of processing some horticultural crops. Boil‐peeling utilizes high‐temperature methods like steam peeling and flame peeling. However, although boil‐peeling is a common method, it has not been extensively studied. Therefore, many uncertainties about the method exist, including its mechanism and its scope of application in peeling fruits.

A positive relationship exists between ethylene concentration and the success of boil‐peeling in the red flesh kiwifruit cultivar, “Rainbow Red” (*Actinidia chinensis* Planch.) (Murakami et al., 2019a). Boil‐peeling in the 100 ppm ethylene‐treated fruits that ethylene produced remarkably were successful; however, in the no‐ethylene treatment were unsuccessful (Murakami et al., 2019a). Furthermore, boil‐peeling was facilitated in persimmon (*Diospyros kaki* Thunb.) and Japanese pear (*Pyrus pyrifolia* (Culta.) Nakai) after ethylene treatment. Additionally, in a previous publication, we reported that boil‐peeling was facilitated in pear (*Pyrus communis* L.), apple (*Malus pumlia* Mill.), avocado (*Persea Americana* Mill), peach (*Prunus persica* L.), ume (*Prunus mume* (Sieb.) et Zucc.), Japanese plum (*Prunus salicina* Lindl.), and loquat (*Eribotrya japonica* Lindl.) after ethylene treatment (Murakami et al., 2019b). However, the mechanism by which ethylene treatment facilitates boil‐peeling in fruits is unclear. Hence, the purpose of this study was to clarify the mechanism by which ethylene facilitates boil‐peeling in fruits. In persimmon, successful boil‐peeling without ethylene treatment has not been reported. Thus, we investigated the effect of ethylene treatment on the peel characteristics of persimmons, that facilitated boil‐peeling using chemical, genomic, and histochemistry analyses.

## 2 MATERIALS AND METHODS

### 2.1 Plant materials

**FIGURE 1:**
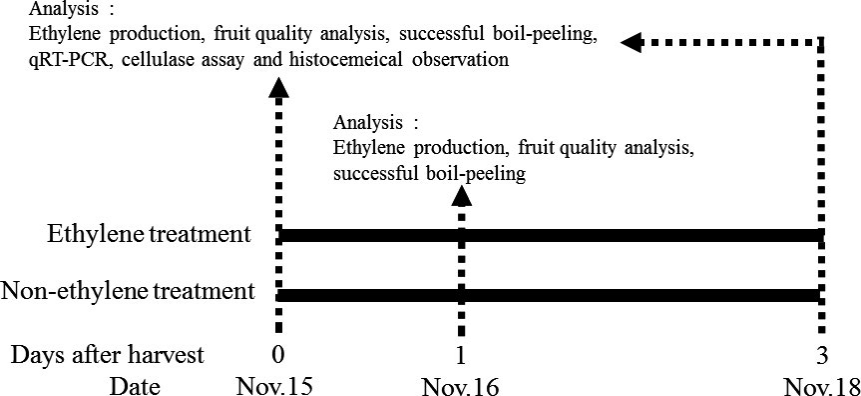
Schematic representation of the experimental design of this study. Experimental fruits were harvested on November 15. The fruits were segregated into two treatment groups; ethylene treatment at 100 ppm for 3 days at 25°C, and the control group which were not treated with ethylene. Fruits were investigated at the points indicated by arrows

The Maekawa‐Jiro persimmons used in this experiment were obtained from an orchard in Shizuoka, Japan. The persimmons were harvested on November 15 and screened to exclude fruits with physical injury, disease, and pest damage. The fruits were assigned into two groups: ethylene‐treated group and the control group. Ethylene treatment was done following the method of Murakami et al. (2019a). Immediately after harvest, the persimmon fruits were treated with 100 ppm ethylene at 25°C for 3 days, while the fruits in the control group were not treated (Figure 1).

### 2.2 Induced ethylene production and fruit quality analysis

Induced ethylene production was evaluated by incubating individual fruits in an 880 ml container for 1 hr, after which 1 ml of headspace gas was withdrawn and injected into a gas chromatograph (GC‐2014). Evaluation of fruit quality was performed using 8 fruits from each treatment. Flesh firmness was determined by measuring compression using a Creep Meter (RE3305) fitted with a 5‐mm diameter plunger. Each fruit was subjected to a compression speed of 1 mm/s. Soluble solid content of the fruit juice was measured using a digital refractometer (DBX‐55A) and the values were expressed in percentage. Titratable acidity (TA) of the fruit juice was determined by titrating the fruit juice against 0.1 N NaOH and it was expressed as percentage citric acid equivalents (% TA).

### 2.3 Quantitative reverse transcription‐polymerase chain reaction (qRT‐PCR)

After ethylene production and fruit quality analysis, approximately 1‐mm thick peel tissue sample was collected from the fruit using a knife. The peel was frozen in liquid nitrogen and stored at −80°C for RNA extraction. Total RNA was extracted from the frozen peel samples using the method for polysaccharide‐rich fruit peel tissues, as described by Ikoma et al. (1996). The samples were treated with DNase (RNeasy; QIAGEN) to eliminate any DNA contamination. First‐strand cDNA was synthesized from total RNA using a reverse transcription kit (Prime Script II 1st strand cDNA synthesis kit; Takara), according to the manufacturer's instructions. The first‐strand cDNA was used as a template for the amplification of cDNA fragments encoding the three ethylene‐related genes in persimmon: 1‐aminocyclopropane‐carboxylate synthase gene (*DK‐ACS1*) (Guinevere et al. 2006), ACC oxidase gene (*DK‐ACO2*) (Guinevere et al. 2006), and polygalacturonase gene (*DKPG1*) (Liu et al., 2009). The sequences of all primers used for qRT‐PCR are listed in Table 1.

**TABLE 1:** Oligonucleotide primers used for amplification of cDNA by qRT‐PCR

Name	Accession	Oligonucleotide sequence
*DK‐ACS1*	AB073005	F:CCTTGAAAGCGCCGGAATTA
		R:TTTCGGAGCTCAAGAGGTGT
*DK‐ACO2*	AB073009	F:TGGAGAGGCTCACAAAGGAG
		R:TTCCCAGTCCAAGTCGTTGA
*DKPG1*	EU816197	F:ACCTCGTCAAGCCAATCAGA
		R:CCAGTGTGTCCCATCTTCCT
*DK‐Actin*	AB219402	F:AACAGGGAGAAGATGACGCA
		R:GTGTGGCTAACACCATCACC

Expressed sequence tags of five ripening‐related genes were obtained from the Kazusa DNA Lab. persimmon database (Kazusa DNA Research Institute) by BLAST analysis (persimmon.kazusa.or.jp/blast.html). Oligonucleotide primers were designed according to the regions of each gene. The specificity of all the primers used was verified by melting curve analysis. qRT‐PCR was performed in a PCR Thermal Cycler Dice^®^ Touch (Takara) using Power SYBR Green PCR (Applied Biosystems), as described in the manufacturer's protocol. Relative expression values were calculated as an average of three independent biological replications using *Dk‐Actin* as the internal standard.

### 2.4 Measurement of cellulase activity

The cellulase (β‐1,4‐glucanase) activity in the persimmon peel was measured following the method of McCleary et al. (2012

) using a commercially available assay kit (Megazyme), according to the manufacturer's instructions. The samples of peel tissue used for the assay were similar to the ones used for qRT‐PCR analysis. The means of three individual experiments were determined from separate but concurrent reactions.

### 2.5 Histochemical observation in peel tissue

Persimmon fruits from each treatment were used for histochemical studies. Tissue samples were obtained via horizontal sections along the equatorial axis of the outer fruit. Collected fruits, including peel tissues, were fixed with 50 mM PBS solution, including 4% paraformaldehyde, and embedded in Technovit^®^ 7100 (Kulzer). The embedded tissues were cut longitudinally to form sections of approximately 10 μm using a microtome. The sections were stained with 0.1% toluidine blue O and 0.01% ruthenium red. Toluidine blue O stains cell wall components, such as pectic substances (O’Brien et al., 1964). Ruthenium red reactions were evaluated by spot testing with 57 substances and by titrating with chemically defined pectins (Luft, 1971). Pictures of the stained sections were taken for subsequent analysis.

### 2.6 Image processing and analysis

**FIGURE 2:**
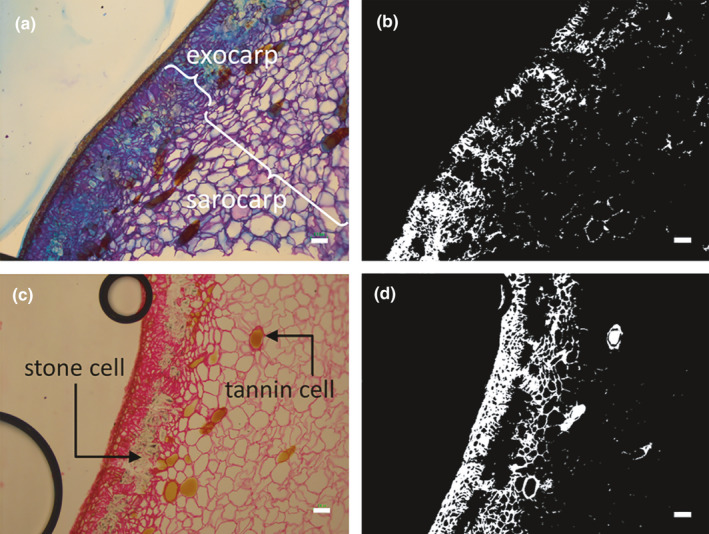
Images of the immunohistochemistry analysis. The images were analyzed to quantify polysaccharides and pectinase in the pericarp. Toluidine blue O (a) and ruthenium red (c) reaction in exocarp and sarcocarp tissue. Picture (b and d) shows the result of the analysis. Bars represent 100 μm

The peel region was divided into two zones: the exocarp and sarcocarp (Figure 2). The intensity of toluidine blue O and ruthenium red staining of the exocarp and sarcocarp were quantified by analyzing images of the section. The images were processed and analyzed using ImageJ software (ImageJ software, NIH) to ascertain the polysaccharide and pectin contents of the exocarp and sarcocarp using the method of Gonzalez et al. (2010). Saturation component images were processed using different filters, and processed images were used to calculate the total cell viability from the intensity of the stained areas of the photomicrograph. The blue component of the RGB (red, green, blue) color image was selected for the toluidine blue O image, and the red component of the image was used for ruthenium red. The threshold was applied to a regular level in toluidine blue O and ruthenium red images. Some of the analyzed figures are shown in Figure 2. The analyzed area of the figure was between 115 and 783 mm^2^_,_ excluding stone cells and tannin cells. A total of nine stained areas with toluidine blue O and ruthenium red were analyzed for staining intensity.

### 2.7 Statistical analysis

The results were analyzed with BellCurve for Excel software (Social Survey Research Information Co., Ltd.) using Tukey's test (*p* < .05) .

**FIGURE 3:**
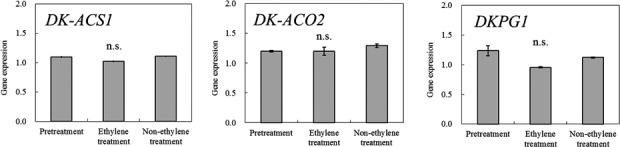
The expression of the ethylene‐related genes, *DK‐ACS1* and *DK‐ACO2*, and pectinase gene, *DKPG,* in ethylene‐treated persimmons and in nonethylene‐treated (control) persimmons. The ethylene‐treated and the control fruits were stored for 3 days at 25°C. Tissues used for the analysis were approximately 1 mm peel tissues. Vertical bars show standard errors of three replicates. n.s. indicates non‐significance at 0.05 level by Tukey's test

## 3 RESULT AND DISCUSSION

**FIGURE 4:**
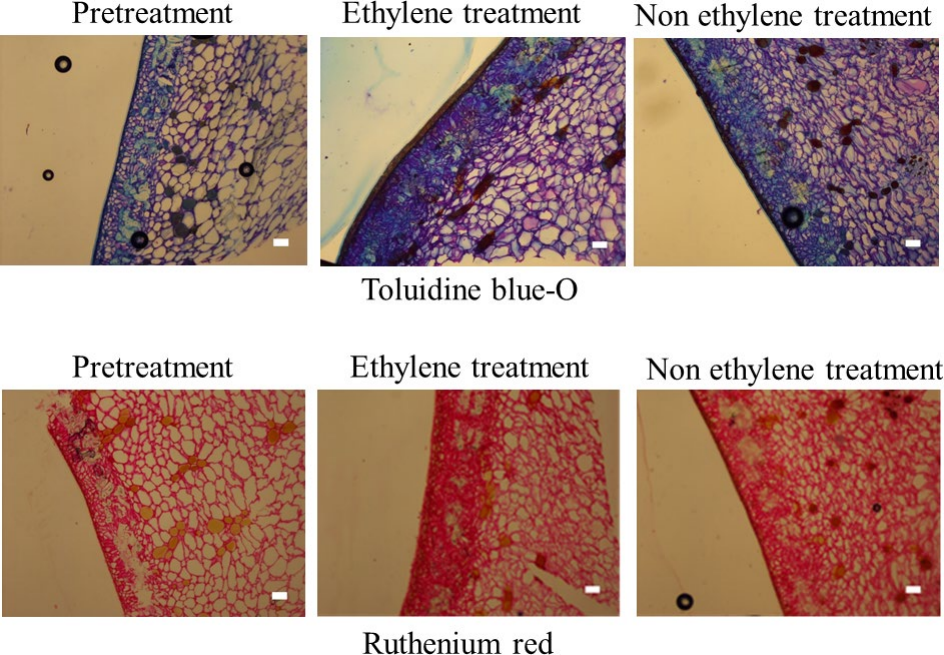
Photomicrographs of the toluidine blue O and ruthenium red reactions in ethylene‐ and nonethylene‐ treated “Maekawa‐Jiro” persimmon pericarp tissues. Scale bars represent 100 μm

**FIGURE 5:**
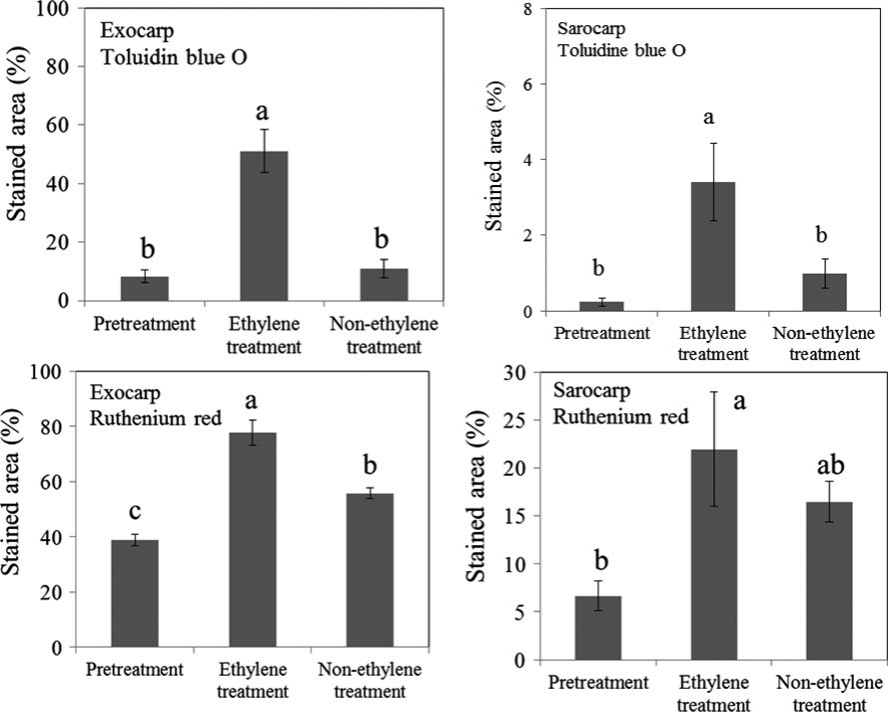
Quantification of stained area as a percentage of total area in “Maekawa‐Jiro” persimmon exocarp and sarcocarp tissue. Vertical bars show standard errors of nine replicates. Bars bearing different letters indicate significant difference between means. Tukey's test was used for mean comparison at *p* < .05 significance level

As compared with ethylene treatment and nonethylene treatment, significant portions of the sarcocarp and exocarp stained by toluidine blue O and ruthenium red in ethylene treatment was thick compared to that of nonethylene treatment, indicating an increase in the quantity of polysaccharides, including pectic substances (Figures 4 and 5). Floros and Chinnan (1988) studied microstructural changes during steam peeling using pimiento pepper (*Capsicum annum* L.). In their study, heat transfer increased the temperature of the fruits, which resulted in several biochemical reactions (i.e., hydrolysis of carbohydrates and pectin breakdown). The results of their study indicated that ethylene treatment increased the quantity of polysaccharides, including pectic substances, directly below the pericarp, which caused the dissolution of the site, resulting in peeling. Ruthenium red staining can be intensified if the pectins are de‐esterified (Benhamou et al., 1990; Marcinowski et al., 1979). For this reason, the increase in staining with ruthenium red may result from de‐esterification of pectin molecule. Thus, pectin methylesterase activity may be important because de‐esterification exposes free carboxy group of galacturnic aid molecules and facilitates the dye to stain pectin.

**FIGURE 6:**
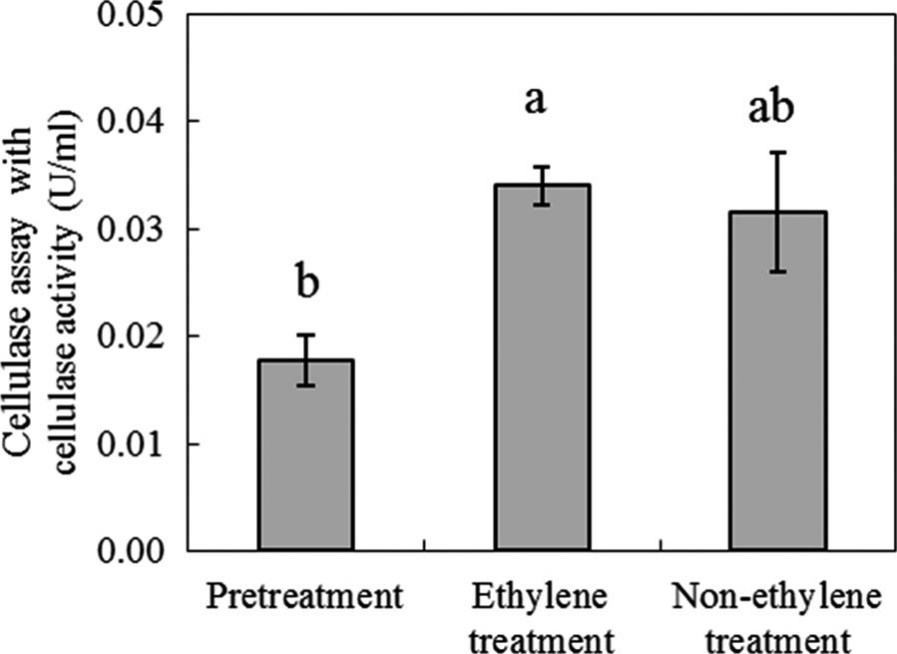
Effect of ethylene and nonethylene treatment on cellulase activity of the pericarp tissue of “Maekawa‐Jiro” persimmon. Vertical bars show standard errors of three replicates. Bars bearing different letters indicate significant difference between means. Tukey's test was used for mean comparison at *p* < .05 significance level

Mauch and Staehelin (1989) studied the subcellular localization of β‐1,3‐glucanase a type of cellulase in ethylene‐stressed bean (*Phaseolus vulgaris* L.) leaves, it found that the small amounts of β‐1,3‐glucanase occurred in middle lamellae of plant cell walls. We investigated the effect of ethylene treatment on the peel characteristics of persimmons by histochemistry analyses using toluidine blue and ruthenium red. However, Benhamou et al. (1990) reported cellulase (β‐1.3‐glucanase) was not stained by toluidine blue. Also, there is no report that ruthenium red stain cellulase. For this reason, we examined the change in cellulase (β‐1,4‐glucanase) activity in the pericarp of persimmon fruits in addition (Figure 6). Cellulase activity increased in ethylene‐treated and in the control samples, indicating that there was no significant difference in cellulase activity between the ethylene‐treated and nonethylene‐treated samples. This suggests that cellulase activity in the pericarp of persimmon may increase with the age of the fruits, and not necessarily as a result of ethylene treatment. Thus, the effect of ethylene treatment on cellulase alone may not be able to facilitate the peeling of the pericarp of persimmons. However, there is still a possibility that native cellulase can act on the cellulose degradation. The method evaluated the cellulase activity in this study does not necessarily reflect the native cellulase activity toward crystalline cellulose. So it is important to study the cellulase activity facilitate the peeling for the detail.

In some crops, ethylene stimulates the synthesis of some enzymes, including polygalacturonase and pectin methylesterase, which results in changes in the cell wall structure (Fischer & Bennett, 1991). Polygalacturonase is one of the hydrolases that destroys cell wall structure (Fischer & Bennett, 1991). In addition, polygalacturonase activity in several fruits increased with an increase in ethylene production, and polygalacturonase activity may be induced in harvested fruit by exogenous ethylene (Saltveit & Mcfeeters, 1980; Sawamura et al., 1978). Therefore, we hypothesized that the increased activity of polygalacturonase by ethylene treatment facilitated boil‐peeling in persimmons in our study (Figure 2, Table 2). However, no significant increase of the pectinase activity, *DKPG* gene was observed in the pericarp of persimmon fruits treated with ethylene. As the reason for this, it was considered the differences analyzed region between histochemical and genomic analysis. In qRT‐PCR, we sampled 1‐mm thick peel tissue. On the other hand, the dyed more deeply region in histochemical analysis was approximately ~400 μm surface layer of the peel (Figure 4). It may be necessary to sample the surface layer of the peel in genetic analysis. Any other factors, such as β‐galactosidase seemed to play a major role in the ripening process of persimmon fruits (Nakamura et al., 2003). Therefore, other factors may be responsible for facilitating boil‐peeling in fruits treated with ethylene.

**TABLE 2:** Fruit characteristics, ethylene production and successful boil‐peeling of ethylene treatment or nonethylene treatment in “Maekawa‐Jiro” persimmon

Treatment	Treatment period	Examined fruits number	Fruit weight (g)	Flesh firmness (g)	Brix (%)	Fruit skin color			Ethylene production (nl/g·hr)	Successful boil‐peeling (%)^a^
						L*	a*	b*		
Ethylene treatment	3	8	241	548	17.5	54.4	31.0	51.5	9.9	100
Non ethylene treatment	3	8	223	2,623	16.1	54.9	31.3	52.1	n.d.^b^	0
t*‐test* ^c^			**	**	n.s.	n.s.	n.s.	n.s.	**
Ethylene treatment	1	8	233	2,445	15.2	58.0	29.1	55.0	13.3	0
Nonethylene treatment	1	8	226	2,663	15.6	58.4	27.2	56.9	n.d.	0
t‐test			n.s.	n.s.	n.s.	n.s.	n.s.	n.s.	**	
Pretreatment	0	8	234	2,575	17.0	58.3	28.8	57.8	n.d.	0

^a^ Successful boil‐peeling fruits number/examined fruits number × 100.

^b^ Not detected.

^c^ n.s.

^**^ Indicate non‐significance or significance at 0.01 levels respectively.

Although ethylene generation was induced in the Maekawa‐Jiro persimmons after the ethylene treatment, the quantity generated was low (Table 1). Furthermore, no significant increase in the expression of the genes responsible for ethylene biosynthesis, *DK‐ACS1* and *DK‐ACO2*, and the gene responsible for pectinase activity, *DKPG*, was observed in the pericarp of persimmon fruits treated with ethylene, and this may be due to the low sensitivity of Maekawa‐Jiro persimmon to ethylene (Figure 3).

Although the increase in the quantity of pectic substances in the persimmons was detected via the histochemistry analysis, the low sensitivity of the persimmons to ethylene made it impossible for any increase in the expression of the pectinase‐related gene to be detected by the qRT‐PCR analysis. In addition, it is also possible that these genes may have been significantly expressed in limited parts of the pericarp.

Once persimmons receive ethylene treatment, they can be peeled with hot water. In the present study, ethylene treatment softened the fruit pulp, but the sugar and acid contents remained the same. Generally, ethylene treatment can cause fruit pulp to soften, which can negatively impact fruit processing. It is, therefore, necessary to conduct studies aimed at optimizing the ideal ethylene treatment conditions.

The use of chemicals, including lye, to peel fruits, has been widely adopted because they are cheap and easy to use (Lucas, 1967). However, the use of lye has several disadvantages, including high peeling losses (Heaton, 1984), allergic reactions (Brenna et al., 2000), and the generation of liquid waste high in NaOH. Alternatively, there is limited information about the mechanism underlying the heat‐peeling method. Li et al. (2014) studied the mechanism responsible for the loosening and cracking of peels under infrared heating in tomatoes and found that it was due to the reorganization of extracellular cuticles, thermal expansion of cell walls, and the collapse of several cellular layers. This study provides new insights on the effect of ethylene on boil‐peeling in persimmons and provides a foundation for future research studying the effect of heat treatment in the peeling of fruits or tomato.

## 4 CONCLUSION

In the present study, we investigated the effect of ethylene treatment on the peel characteristics of persimmons, that facilitated boil‐peeling, using chemical, genomic, and histochemistry analyses. As compared with ethylene treatment and nonethylene treatment, significant portions of the sarcocarp and exocarp stained by toluidine blue O and ruthenium red in ethylene treatment was thick compared to that of nonethylene treatment, indicating an increase in the quantity of polysaccharides, including pectic substances. The ethylene‐related genes, *DK‐ACS1* and *DK‐ACO2*, and the pectinase‐active gene, *DKPG*, were not significantly expressed in the pericarp of persimmons, even though a small amount of ethylene was generated after ethylene treatment. The study showed that boil‐peeling in persimmons was possible because ethylene treatment increased the quantity of pectic substances in the pericarp of the fruit which resulted in the softening of the peel. This study provides new insights on the effect of ethylene on boil‐peeling in persimmons and provides a foundation for future research studying the effect of heat treatment in the peeling of fruits or tomato.

## CONFLICT OF INTEREST

The authors declare that there are no conflicts of interest.

## ETHICAL STATEMENT

This study does not involve any human or animal testing.

## Data Availability

Data available on request from corresponding author.
